# Opportunities of predictive medicine at the treatment of diabetes mellitus complications

**DOI:** 10.1186/1878-5085-5-S1-A73

**Published:** 2014-02-11

**Authors:** Maxim Sautin, Sergey Suchkov

**Affiliations:** 1 European clinic of sports traumatology and orthopaedics (ECSTO), Moscow, Russia; 2 Moscow State Medical & Dentistry University, Moscow, Russia

## 

Foot ulcers develop in approximately 15 percent of type 1 Diabetes patients. 85% of the lower-limb amputations in those patients are preceded by foot ulceration, suggesting that prevention and appropriate management of foot lesions are of marked value. Ulceration is caused by several factors acting together, but particularly by neuropathy. The annual incidence of foot ulceration is slightly larger than 2.0 percent among all T1D patients and between 5.0 and 7.5 percent among T1D patients with peripheral neuropathy [1].

Peripheral neuropathy results in a loss of the protective sensation of pain and in autonomic dysfunction, with sympathetic denervation, dry skin, and warm feet. Appropriate medical education regarding early assessment for lesions or warning signs of imminent ulceration in patients with sensory loss is essential for the future to come. Other important component causes of ulceration include peripheral vascular disease, callus, edema, and deformity. The triad of neuropathy, deformity, and trauma is present in almost two thirds of patients with foot ulcers. Inappropriate footwear is the most common source of trauma [2].

The economic burden associated with diabetic foot ulceration, i.e., a condition that is preventable in many cases, is enormous. The estimated cost of treating one foot ulcer over a two-year period is $28,000. Growth Factors Recombinant platelet-derived growth factor was the first growth factor approved by FDA for the treatment of neuropathic foot ulcers in T1D patients. A recent review of growth factors in the treatment of diabetic foot ulcers concluded that, platelet-derived growth factor may be useful in chronic, non healing europathic ulcers that do not respond to conventional care [3].

Tissue-engineered skin comprises a cultured living dermis and sequentially cultured epidermis, the cellular components of which are derived from neonatal foreskin. Treatment with tissue-engineered skin is associated with faster healing and lower rates of osteomyelitis and lower-limb amputation [4].

Alternative insulin therapies are being sought that will provide glycemic control for people with diabetes mellitus. The epidermis is a self-renewing tissue that is easily accessible and can provide large numbers of autologous cells that can be used for generating insulin-secreting skin substitutes. Lentiviral vectors have been engineered to produce a fusion protein between the furin-cleavable proinsulin and the self-dimerization mutant of FK506-binding protein to yield bioactive insulin in keratinocytes; this insulin is released as a response to exogenous administration of a small organic molecule, rapamycin [5].

The bioengineered keratinocytes retained normal morphology and grew in a manner similar to lentiviral-treated control cells. There are a lot of products of bioengineering, different scaffolds, which can be used as base for tissue regenerating. From our point of view, one of the main points of preventive effect in the treatment of diabetes is to secure early accumulation of the personalized genetic information about the patient. This is necessary not only for treatment of the underlying disease, but treatment of its complications. Modern technologies allow creating biomaterials that can provide not only local, but also the overall therapeutic effect when they are implanted in the area of ulceration in diabetes.
